# The Impact of Digital Economy on Residents' Health: Based on the Perspective of Population Ageing

**DOI:** 10.3389/fpubh.2021.725971

**Published:** 2021-07-26

**Authors:** Zhi-Ting You, Min Zhong, Qing Gao, Hong-Xiang Wei, Xi-Hao Zeng

**Affiliations:** ^1^Business School, Guangxi University, Nanning, China; ^2^College of Economics, Jinan University, Guangzhou, China; ^3^Graduate School, Nanning Normal University, Nanning, China; ^4^College of Economics and Management, Nanning Normal University, Nanning, China; ^5^College of Economics and Management, Nanning Normal University, Nanning, China

**Keywords:** digital economy, resident' health, population ageing, fixed effect regression model, panel threshold regression model

## Abstract

This paper uses panel data from inland provinces of China to perform a fixed effect regression and finds that the development of the digital economy has a significant promotional effect on the health of residents. Then, the population ageing rate is further used as a threshold variable for a threshold regression, and the relationship between the development of the digital economy and the health of residents from the perspective of ageing is discussed. The empirical results show that the ageing of the population will reduce the role of the digital economy in promoting residents' health. There is a non-linear single threshold effect between the development of the digital economy and residents' health indicators. In areas of China with a better developed digital economy, when the ageing rate exceeds the threshold, the positive impact of the development of the digital economy on population health has increased compared with the population ageing below the threshold. These asymmetric developments are closely related to economic development, historical and cultural factors, and policies formulated by the government. Therefore, as the digital economy continues to advance, the government should also provide health services fairly and efficiently, and formulate effective Internet assistance policies for the elderly so that the development of the digital economy can more comprehensively promote the health of residents of all ages.

## Introduction

**Figure 1:**
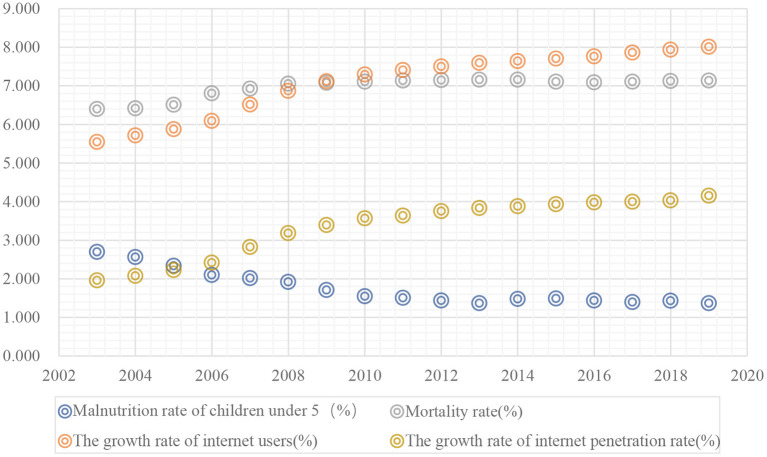
The impact of the digital economy on residents' health.

This paper aims to explore whether the development of the digital economy (measured by the growth rate of Internet users and the Internet penetration rate) will affect the health of residents (measured by the malnutrition rate of children under 5 years old) under the general background of population ageing, and whether there will there be structural changes in the effect of the development of the digital economy on the health of residents as the ageing of the population intensifies. With the rapid development of new-generation technologies such as the Internet, cloud computing, and big data in various fields (1), human society is entering a new historical stage marked by digital productivity. The concept of the digital economy was formally proposed by “The Emerging Digital Economy” report issued by the U.S. Department of Commerce in 1998. Different organisations and countries combined their own external environment and other factors to further interpret the digital economy. The OECD believes that the digital economy is based on electronics commodities and trade services for business. In 2019, China's digital economy totalled 3.58 billion yuan, accounting for 36.2% of the country's total GDP; and it has become one of the important engines of China's economic development. With the improvement of residents' living standards and the intensification of the ageing population, the problems of “difficult to see a doctor” and “expensive treatment” are common worldwide. In order to obtain health information, prevent diseases and receive medical services more conveniently, Chinese residents have a high acceptance of using platforms such as the Internet to seek medical treatment. The 43rd Statistical Report on Internet Development in China shows that 19.2% of Chinese non-real-name users are willing to use the Internet to obtain professional medical information and services. Figure 1 shows the relationship between the development of the digital economy and the health of residents. Figure 1 shows that there is an obvious “scissors gap” between the rate of severe malnutrition of children under 5 years old, which measures the health of residents; and the growth rate of Internet penetration, which measures the development of the digital economy. The relationship between the mortality rate and the growth rate of Internet users is similar, which indicates that there may be a significant positive correlation between the development of the digital economy and the health of residents. The application of big data technology can more accurately locate the health care needs of residents through data analysis. The combination of platforms such as the Internet and those used by medical and health industries can improve the efficiency and reduce the costs of medical treatment. The rapid development of the Internet of Things and other fields guarantees the timely distribution of subsequent medical products, and the health of residents can obtain systematic, full-cycle, and customised services, thereby improving the health of residents (2, 3).

Population ageing is an important trend in social development and a global phenomenon. In 2019, China's ageing rate was 11%, and the dependency ratio of the elderly population had increased to 19.6%, which means that one elderly person needs five working-age people to bear the burden. Furthermore, the ageing rate in developed countries such as Japan, Germany, and the United States has reached 27, 21, and 15%, respectively. The ageing trend of the population is putting increasing pressure on the medical and health system, especially in developing countries where the economy is in urgent need of development. Generally, the medical system in developing countries is more fragile than in developed countries, and the development of the digital economy is facing technical bottlenecks. Therefore, it is more difficult to promote the health of residents through digital economic development in developing countries than in developed countries. However, the successful application of the digital economy in the field of health and hygiene in developing countries will theoretically achieve more utility than in developed countries. First, the elderly can learn about and obtain professional medical services and products through platforms such as the Internet and the Internet of Things, thereby breaking the monopoly of medical institutions. Second, technologies such as big data, cloud computing, and the Internet are more powerful than traditional algorithms (4), and can more accurately locate the physical and psychological needs of the elderly. It is conducive to the elderly to acquire health care knowledge and participate in social interaction (5), thereby increasing their health knowledge and alleviating their loneliness, which is conducive to the improvement of the health of the elderly. Furthermore, the elderly no longer have to go to the hospital for minor illnesses, which can effectively save social resources. Therefore, the combination of digital economy platforms such as the Internet and health and sanitation industries can effectively strengthen the medical system in developing countries.

Like nature (6), technology, culture and other factors, the health of residents as a measure of population quality is an important prerequisite for national economic stability. Although there is a relatively intuitive and positive relationship between the development of the digital economy and the health of residents in the context of population ageing, it cannot be ignored that the Internet, as one of the main platforms of the digital economy, will cause sleep deprivation among people who are excessively addicted to the it and negatively affect the bodies of people with suboptimal-health (7). Therefore, does the rapid development of the digital economy have a statistically significant positive impact on the health of residents? What is the impact? In addition, in a sample from China, as the ageing of the population intensifies, is there any structural change in the impact of the development of the digital economy on the health of residents? Based on this, the marginal contributions of this paper are follows. First, based on the empirical results of Chinese samples, it is found that the digital economy has a significant positive impact on the health of residents. Second, in the context of population ageing, the positive impact of the development of the digital economy on the health of residents has declined, which indicates that the elderly has insufficient use of digital economy related products due to the solidification of living habits and the decline in learning ability. Third, as the population ageing process continues to intensify, there are structural changes in the impact of digital economic development on the health of residents, and regions with different economic development levels and digital economic development levels have different impact sizes and structural changes. This mainly shows that the higher the level of economic development and the better the development of the digital economy are, the more obvious the promotional effect of the region.

The structure of the remainder of this paper is as follows. Section Literature Review reviews the existing literature and section Health Capital and the Demand for a Health Model With a Digital Economy uses the demand for the health model to briefly describe the theoretical mechanism. Section Methodology introduces the empirical method. Section Data shows empirical data and variables. Section Empirical results analyses the empirical results. Section Conclusions is the concluding remarks.

## Literature Review

The digital economy refers to the realisation of the recognition, selection, filtering, storage and use of digital knowledge and information based on the Internet and other network infrastructures, so as to realise the economic form of high-quality economic development. The development and popularisation of the digital economy are the trend and inevitable result of scientific and technological progress. Many scholars at home and abroad have discussed the relationship between the development of the digital economy and the health of residents and have mainly formed the three major viewpoints of health promotion, technical pressure and indirect relationship. Among these viewpoints, the theory of health promotion believes that users of digital economy technology tend to have better physical and mental health and medical decision-making capabilities. Hong et al. (8) and Wangberg et al. (9) found that Internet use has a significant positive effect on self-rated health. Tasi et al. (10) found that residents who frequently use the Internet have a lower probability of suffering from depression. Minto et al. (11) and Sami et al. (12) found that frequent Internet use can relieve anxiety in patients with cardiomyopathy. Technological stress theory refers to adaptive diseases caused by the inability to handle Internet technology in a healthy way (13), such as Internet addiction. Allcott et al. (14) detected that excessive Internet use will increase the health risks of users. Long-term Internet use can lead to anxiety (15), a lack of sleep (16) and depression (17); it is particularly serious in Asian regions such as South Korea and China (18, 19). Indirect relationship theory believes that the Internet and other network infrastructures are self-selected by users and that use of the Internet and other platforms actually reflects the role of socioeconomic education and does not directly affect health (20). Most of the views in the existing literature tend to be that the development of the digital economy plays a role in promoting the health of residents. The view of technological pressure can be summarised as the rational use of technology platforms such as the Internet or the degree of use. Everything has two sides, and digital development is obviously no exception. The rational use of the convenience brought by the development of the digital economy will help promote the health of residents.

According to the existing literature, the mechanism of the impact of the development of the digital economy on the health of residents is roughly investigated from the perspectives of information acquisition and interpersonal communication. The information acquisition mechanism means that the development of the digital economy provides diversified and accessible platforms for the acquisition of health information. Residents can inquire about health knowledge through the Internet, complete medical treatment through online contactless consultation, participate in offline drug delivery, etc. to improve their health. According to McMullan's research (21), platforms such as the Internet have effectively broken the monopoly of offline medical institutions on professional information, alleviated the information asymmetry between doctors and patients, and are more conducive to residents' management of their own health. Mano (22) found that residents' online health services through the Internet can improve personal health literacy and strengthen personal health management. Sillence et al. (23) also put forward that users of platforms such as the Internet can improve their health through channels such as searching for disease information and participating in online health activities. The interpersonal communication mechanism refers to the social activities, leisure and entertainment of residents through platforms such as the digital economy, which is conducive to alleviating personal loneliness, depression or anxiety of residents, and promoting residents' mental health. Boneva and Kraut (24) took a community in Toronto, Canada as an example. The study found that residents who use the Internet know more than three times as many neighbours, are twice as likely to meet neighbours, and are 1.5 times more likely to visit neighbours than those who do not use the Internet. Therefore, Internet users can better connect with neighbours. Lagoe and Atkin (25) used structural equation modelling to study the relationship between Internet use and mental health with 245 American adults as the research objects. The study showed that using the Internet can reduce stress and reduce levels of depression and loneliness. Szabo et al. (26) using a combination of longitudinal mediation analysis and demographic control, through a survey of 165 adults aged 60–77, found that the Internet can alleviate the loneliness of the elderly through three channels: social interaction, tools, and information.

Due to changes in the age structure of residents, the intensification of population ageing will have an impact on the relationship between the digital economy and residents' health. People in different eras have different acceptance capabilities for the digital era (27). The “digital natives” born in the digital age are more familiar with and integrated into the digital age than the “digital immigrants” born in the digital age. Before elderly people were called “digital immigrants” are called “digital refugees” (28). Older people are difficult to integrate into the digital age due to their inherent habits and degraded learning ability; however, they are the group most in need of medical services. However, compared with adolescents, middle-aged and elderly people have stronger self-control abilities and are better at using digital economy platforms to obtain useful health information, strengthen their communication with society, and reduce their loneliness. Furthermore, using these platforms can improve the physical and psychological health of the elderly (29–31).

In summary, the research results based on different countries and different perspectives show that the development of the digital economy mainly has a positive impact on the health of residents, and it is roughly transmitted through two channels: information acquisition and interpersonal communication. However, in the context of population ageing, whether the impact of the development of the digital economy on the health of residents is underestimated and whether there are structural changes are still rarely studied in the literature. Therefore, this paper will discuss the above issues in depth.

## Health Capital and the Demand for a Health Model With a Digital Economy

In the study of economics, health can be regarded as an important part of human capital. Health investment depends on the return and cost of an investment. The returns include health as a consumer product that can increase the utility of investors (good health can be satisfied) directly into the utility function and as a capital good (good health can be included in labour) affecting the total time used for market and non-market activities. The costs include the time spent to obtain health; the costs of elements such as purchased food, entertainment, and medical services; and the costs caused by the impact of specific environmental variables, such as education level, which can be regarded as an intangible production technology and will affect the efficiency of the healthy production process. Whether to invest in health depends on the size of the benefits and costs. If the benefits exceed the costs, more health investment will be made.

With the development of information technology such as the digital economy, the digital economy not only affects people's production and lifestyles but also has an increasingly deeper impact on health investment (32). Therefore, this paper uses the demand for health to model and systematically analyse the effect of the development of the digital economy on health demand (33).

In Grossman's demand for health model, the utility function of consumers in their lifetime is set as *U*(*H, Z*), where *H* refers to the health of consumers. *H* is related to medical services (*M*), the time consumers spend on health production (*T*_*h*_), and other factors (*E*) such as education level, etc. *Z* refers to other consumer goods that can obtain utility. *Z* is related to the number of other consumer products (*X*), the time spent by consumers to produce other consumer products (*T*_*Z*_), and other factors (*E*). The health utility function of the consumer's lifetime can be expressed as follows:

(1) $$\begin{array}{l} U=U\left(\varphi_{0}H_{0},\cdots \varphi_{t}H_{t}\cdots \varphi_{n}H_{n},Z_{0},\cdots \;,Z_{t},\cdots Z_{n}\right) \end{array}$$

(2) $$\begin{array}{l} H=G_{1}\left(M,T_{h}\;;E\right) \end{array}$$

(3) $$\begin{array}{l} Z=G_{2}\left(X,T_{h},\;T_{z}\;;E\right) \end{array}$$

*H*_0_ represents the initial stock of healthy capital, *H*_*t*_ represents to the consumer's stock of healthy capital in period *t*, φ_*t*_ represents the return per unit of health capital, φ_*t*_*H*_*t*_ represents health consumption in period *t*, and *Z*_*t*_ represents the total consumption of other commodities in period *t*. In this model, the consumer's life cycle length *n* is endogenous, the health capital stock *H*_*t*_ is also endogenous, and the initial health capital stock *H*_0_ is an exogenous variable. The increase in health capital is:

(4) $$\begin{array}{l} H_{t+1}=\left(1-\delta_{t}\right)H_{t}+I_{t} \end{array}$$

Here, *H*_*t*+1_ represents the health capital stock of consumers in period *t* + 1; *H*_*t*_ represents the health capital stock of consumers in period *t*; *I*_*t*_ represents represents the health capital investment of consumers in period *t*; and δ_*t*_ refers to the health depreciation rate, which is determined by external factors and changes as consumers age. The development of the digital economy provides a higher level of medical services. Consumers can obtain online medical services quicker and more conveniently through various Internet platforms. Manufacturers can accurately recommend drugs and health products to consumers based on big data calculations, improve consumer health investment in the current period and postpone the depreciation of previous consumer health capital to enhance consumer utility. Using the form of the healthy depreciation rate proposed by Cropper in 1981 (34), δ_*t*_ can be expressed as:

(5) $$\begin{array}{l} \delta_{t}=\delta_{0}{{\;D}_{t}}^{\alpha }\;O_{t}^{\beta } \end{array}$$

where δ_0_ refers to the initial health depreciation rate; *D*_*t*_ represents the development level of the digital economy (negative); and *O*_*t*_ represents other factors that affect the health depreciation rate, such as whether to drink alcohol or stay up late. The health investment function *I*_*t*_ can be expressed as:

(6) $$\begin{array}{l} I_{t}={{\;D}_{t}}^{\alpha }\;M_{t}^{\gamma }\;GO_{t}^{\omega }\;E_{t}^{\rho } \end{array}$$

Here, *M*_*t*_ is the medical services that consumers can purchase, *GO*_*t*_ is the public services provided by the government, and *E*_*t*_ is other influencing factors such as education level and labour income. Substituting Equations (5) and (6) into Equation (4), we can obtain:

(7) $$\begin{array}{l} H_{t+1}=\left(1-\delta_{0}{{\;D}_{t}}^{\alpha }\;O_{t}^{\beta }\right)H_{t}+{{\;D}_{t}}^{\alpha }\;M_{t}^{\gamma }\;GO_{t}^{\omega }\;E_{t}^{\rho } \end{array}$$

By calculating the partial derivative of *D*_*t*_, we can obtain:

(8) $$\begin{array}{l} \frac{\partial H_{t+1}}{\partial D_{t}}=\alpha {D_{t}}^{\alpha -1}\left(M_{t}^{\gamma }\;GO_{t}^{\omega }\;E_{t}^{\rho }-\delta_{0}{{\;D}_{t}}^{\alpha }\;O_{t}^{\beta }H_{t}\right) \end{array}$$

It equation shows that the impact of the level of digital economic development on health investment is also affected by the health services *M*_*t*_ that consumers can purchase, the public services *GO*_*t*_ provided by the government, and other influencing factors.

The budget constraints faced by consumers throughout their lives are:

(9) $$\begin{array}{l} \;\Sigma \left[\left({\;P}_{t}\;M_{t}+V_{t}X_{t}\right)/{\left(1+r\right)}^{t}\;\right]\\ =\Sigma \left[\left(W_{t}Tw_{t}\;/{\left(1+r\right)}^{t}\right)\right]+A_{0} \end{array}$$

(10) $$\begin{array}{l} Tw_{t}+TI_{t}+T_{h}+T_{Z}\;=\Omega \end{array}$$

Here, Equation (9) is the income constraint, and Equation (10) is the time constraint. In (9), *P*_*t*_ and *V*_*t*_ represent the market prices of *M*_*t*_ and *X*_*t*_, respectively; *r* is the interest rate; and *W*_*t*_ is the wage rate (determined by external factors) for a consumer in period *t*. *Tw*_*t*_ is the time consumers spend working in period *t*, *A*_0_ represents initial wealth, *TI*_*t*_ represents the time lost when consumers are unable to engage in market or non-market activities due to illness (hereafter referred to as the time of illness), and Ω is the number of days for consumers in each period (e.g., if one period is equal to 1 week, then Ω is equal to 7 days).

The above 10 formulas constitute the consumer's health demand model. Consumers will make the best choice related to their health demand under the conditions of budget and time constraints to maximise their utility. The equilibrium conditions based on this model are:

(11) $$\begin{array}{l} \left[\frac{W_{t}}{\pi_{i-1}}+\frac{\left(U_{ht}/m\right){\left(1+r\right)}^{i}}{\pi_{i-1}}\right]\;G_{t}=r+\delta_{t} \end{array}$$

**Figure 2:**
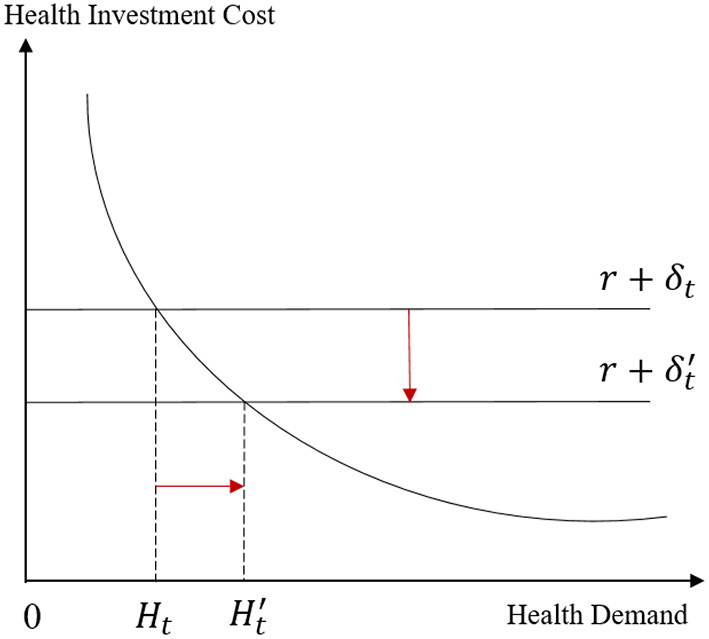
Balanced analysis of health demand.

Here, *G*_*t*_ = ∂*TI*_*t*_/∂*H*_*t*_ represents the marginal output of health, *U*_*ht*_ = ∂*U*/∂*H*_*t*_ represents the marginal utility of health, *m* represents the marginal utility of money, and π_*i*−1_ represents the marginal cost of investing in health. The return on health investment is composed of *W*_*t*_*G*_*t*_/π_*i*−1_ and $\left(U_{ht}/m\right){\left(1+r\right)}^{i}G_{t}/\pi_{i-1}$. The cost of health investment is composed of interest rate *r* and health depreciation rate δ_*t*_, and the equilibrium conditional equation (9) indicates that when the marginal benefit of health and the marginal cost are equal, the consumer maximises their utility. The optimal health demand *H*_*t*_ is determined by the intersection of the marginal revenue curve and the marginal cost curve (Figure 2).

On the cost side, if the depreciation rate δ_*t*_ in the cost of health investment decreases, it will definitely move the marginal cost curve downward, leading to an increase in health demand. On the income side, if the marginal cost of health investment π_*i*−1_ decreases, health demand will move down the demand curve, which will also lead to an increase in health demand. Combining formulas (5) to (7), the development of the digital economy can affect the demand for health by influencing the depreciation rate and current health investment under budget constraints. Furthermore, the existing literature shows that the Internet has become an effective tool for disseminating health information and preventing diseases, breaking the monopoly of professional health information by medical institutions; patients can use the Internet and other platforms to obtain health services (1), such as remote registration, remote diagnosis and treatment, and light consultations (35), across time and spatial barriers, thereby improving the efficiency of health investment and saving medical treatment costs. On this basis, the following hypothesis is proposed:

Hypothesis 1: Under the control of other factors, the development level of the digital economy has a significant positive effect on the health of residents.

Furthermore, regarding age, young people's self-control ability is poor, and they tend to indulge in various entertainment activities provided by the digital economy and damage their health. Middle-aged people have more self-control than teenagers. Middle-aged people can make better use of the digital economy to invest in health. The use, proficiency and acceptance of the digital economy by young and middle-aged people is greater than that of the elderly. Elderly people have barriers to applying the digital economy, and their use of the digital economy to improve their health will be lower (36). The demand for health model also reflects that as age increases, the cost-side health depreciation rate will rise. In other words, as age increases, the health status and health capital stock of a country's residents will decline (37). As China's ageing problem has intensified, ageing has become the most important risk factor affecting the quality of life of Chinese residents. On this basis, the following hypothesis is proposed:

Hypothesis 2: Residents with different characteristics use the digital economy to improve their health to different degrees. Compared with young people and middle-aged people, the extent to which the elderly can improve their health through the digital economy will be weaker, that is, the ageing problem will greatly reduce the impact of the digital economy on improving the health of residents.

## Methodology

In order to test the impact of the development of the digital economy on the health of residents, the following benchmark regression model was constructed:

(12) $$\begin{array}{l} denutri_{it}=\theta_{0}+\theta_{1}{Ln\text{\_}inpeo}_{it}+\sum_{i}\theta_{i}Control+\mu_{i}+\varepsilon_{it} \end{array}$$

Here, *denutri*_*it*_ is the health level of residents (measured by the malnutrition rate of children under 5 years old), and *ln*_*inpeo*_*it*_ is the level of digital economic development (measured by the number of people surfing the Internet). *Control* represents the set of control variables, including government fiscal revenues (*ln*_*govinc*), government fiscal expenditures (*ln*_*govoutc*), per capita years of education (*ln*_*yedu*), total labour wages (*ln*_*labinc*), and total amount of imports and exports (*ln*_*imaex*) in this paper. θ_0_ represents the constant term, θ_1_ is the coefficient of the explanatory variable of the level of the digital economy, and θ_*i*_ is the coefficient of the control variable (*Control*). μ_*i*_ represents the individual heterogeneity, and ε_*it*_ represents the random disturbance term.

On the basis of the above regression model, based on the derivation and hypothesis of the mathematical model, we also need to consider that as the ageing of the population intensifies, the development level of the digital economy may show differences in the impact of residents' health (38). Therefore, this paper uses threshold effect regression to proceed. For further analysis, this paper considers the ageing rate of the population (*oldrate*) as a threshold variable to construct a model:

(13) $$\begin{array}{r} denutri_{it}=\theta_{0}+\theta_{11}{Ln_{inpeo}}_{it}I\left(oldrate_{it}\leq \eta_{1}\right)\\ +\theta_{12}{Ln_{inpeo}}_{it}I\left(\eta_{1}<oldrate_{it}\leq \eta_{2}\right)+\ldots \\ +\theta_{1\;n+1}{Ln_{inpeo}}_{it}I\left(\eta_{n}\leq oldrate_{it}\right)\\ +\sum_{i}\;\theta_{i}Control+\mu_{i}+\varepsilon_{it} \end{array}$$

Here, the dependent variable, independent variables and control variable of formula (13) are the same as those in formula (12). η_*n*_ represents a specific threshold value. The age structure is divided into different intervals through the threshold value. *I*(·) is an indicative function. If the conditions in parentheses are met, then the value is 1. Before performing the threshold panel model regression, *bootstrap* testing is required to set the threshold. This paper uses 300 samplings. First, a single threshold test is performed on the data. If the test is failed, it proves that the relationship between the level of digital economic development and the health of residents will not change in structure as the ageing of the population increases; conversely, if the test is passed, the data can continue to *bootstrap* testing with double thresholds, inspection, and other analysis steps.

## Data

This paper uses the annual data of 30 provinces and cities in inland China from 2003 to 2019 (because there is a considerable amount of missing data in Tibet, it is removed) and a total of 510 samples. Since entering the 21st century, China's digital economy has gained rapid popularity and development. Considering the availability of data, the starting year of the data is set to 2003. The data come from the “China Statistical Yearbook” and the “Statistical Bulletin of National Economic and Social Development” of various provinces, municipalities and autonomous regions.

Judging from the existing research, the evaluation of the health level generally includes both subjective and objective aspects. Subjective standards include self-evaluations of the level of health. Objective standards include medical standards evaluated by doctors, such as nutritional intake, and functional standards evaluated by prevalence. Scholars mostly use mortality to measure the health level of residents in a country or a region, but this method has certain limitations; it ignores the influence of factors such as the age structure of the population in a region, and it cannot reflect the physical and living conditions of the people who are surviving. In order to better reflect the health level of residents, this paper uses the malnutrition rate of children under 5 years old as the explained variable. Children are the future of a country and nation, and indicators that reflect the health of children can also reflect the living conditions of the family. The malnutrition rate of children under 5 years old is used to measure the health of residents and is more representative than the mortality rate. This indicator is an inverse indicator. That is, the higher the malnutrition rate of children under 5 years of age is, the worse the health of residents. This paper intends to use the number of Internet users as a variable to measure the development of China's digital economy and use the Internet penetration rate as a robustness test. Internet use is a foundation for the development of the digital economy, information dissemination, and big data. Therefore, the number of people surfing the Internet and the penetration rate can better reflect the development of China's digital economy. The higher the penetration rate of the Internet is, the better the development of the digital economy in the region.

This paper incorporates five control variables, including government revenues, government expenditures, per capita years of education, labour compensation, and total imports and exports. The first control variable is government revenues. Government revenues can measure the financial strength of the government in the area and can reflect the scope and quantity of the public goods and services provided by the government in social and economic activities. While economic activities will have a significant impact on the health of residents, such as urbanisation (39). The second is the government expenditure. The quality of the government's system can effectively coordinate regional economic activities and non-economic activities (40). Government expenditures can reflect the government's control and use of social resources through the flow of fiscal funds. Government expenditures include various subjects such as health, education, science and technology expenditures. Generally, the more the government spends on health, the more convenient the medical services that residents can obtain are, the more the government spends on education, and the greater the number of opportunities for residents to obtain more educational opportunities; residents who have received more education value their own health more and have more ways and channels to improve their health. The development of science and technology can provide residents with a more convenient and quick life. In summary, government expenditures will affect people's health. The third control variable is per capita years of education. Grossman believes that the improvement of the education level will improve the efficiency of people's production and health so that people's actual health capital stock will also increase (41). The fourth control variable is labour compensation. Labour compensation, that is, income, is the factor that can affect the living standards of labourers. Generally, the higher the labour compensation is, the more medical services a person is willing to consume when facing health problems. Therefore, the residents in an area with higher incomes are more likely to have high health levels. The fifth control variable is the total imports and exports. The foreign trade in a region can reflect the degree of openness of the region. The more open a region is, the higher the degree of economic development, and the more that residents in the region are willing to invest in health. In order to ensure the accuracy of the measurement results, this paper has performed logarithmic processing of the above indicators.

Table 1 shows the descriptive statistics of the main variables. The table shows that there is a large gap in the malnutrition rate of children under 5 years old among various provinces in China, with the minimum value being 0.06 and the maximum value being 16.6600. There is a regional gap in the number of Internet users in various provinces, and there is still a large gap between the maximum and minimum after taking the logarithm, which may be related to the difference in the base of the permanent population in each region. There are also large differences in the degree of Internet penetration in various regions, and the gap between the minimum value and the average value is large. This may be due to the limited Internet access conditions and low Internet penetration in some areas with relatively backward economic development.

**Table 1:** Descriptive statistics of the variables.

**Variables**	**Obs**	**Mean**	**Std. Dev**.	**Min**	**Max**
Denutri	510	2.0435	2.0246	0.0600	16.6600
ln_inpeo	510	6.8541	1.1513	2.9957	9.2587
ln_inrate	510	3.2700	0.8772	0.7655	4.5049
Oldrate	510	9.6757	2.1267	5.4317	16.3751
ln_govinc	510	16.4902	1.1247	12.6126	21.4725
ln_govoutc	510	16.8725	0.9853	13.8681	18.8736
ln_yedu	510	2.1610	0.1162	1.7984	2.5432
ln_labinc	510	8.4960	1.0424	5.3504	10.7321
ln_imaex	510	17.1476	1.7068	12.7342	20.8109

## Empirical Results

In order to avoid the emergence of a “spurious regression” and ensure the accuracy of the estimation results, it is usually necessary to analyse the stationarity of the panel data, that is, to detect whether the data are stable by a unit root test. In order to make the test results have strong robustness and persuasiveness, this paper adopts the *LLC* test for the hypothesis of homogeneous panels and the *IPS* test for the hypothesis of heterogeneous panels at the same time. The *LLC* test allows different intercept terms and time trends, heteroscedasticity and higher-order serial correlation (42). *IPS* inspection is extremely sensitive to the setting of restrictive trends (43).

The test results are shown in Table 2. In general, whether it is a full sample or subsample unit root test, the null hypothesis of unit roots is rejected for all variables at the 10% significance level in both tests. It can be considered that the panel data in this paper are stable, so we can proceed to the next analysis.

**Table 2:** Panel unit root tests.

**Variables**	**LLC unit-root test**		**IPS unit-root test**	
	**Adjusted *t***	***p*-value**	**Z-t-tilde-bar**	***p*-value**
Denutri	−8.3011	0.0000	−12.0777	0.0000
ln_inpeo	−6.8150	0.0000	−5.7880	0.0000
ln_inrate	−5.3087	0.0000	−4.9242	0.0000
Oldrate	−6.2425	0.0000	−5.1393	0.0000
ln_govinc	−11.2206	0.0000	−5.3518	0.0000
ln_govoutc	−13.4622	0.0000	−8.7788	0.0000
ln_yedu	−4.7824	0.0000	−7.4606	0.0000
ln_labinc	−14.5094	0.0000	−6.1318	0.0000
ln_imaex	−4.1112	0.0000	−5.2395	0.0000

### Benchmark Regression Results

Before the regression analysis, it is necessary to pass the *LM* test and the *Hausman* test and select the most appropriate model from the three benchmark regression models of the panel data: the mixed regression model, the random effects model and the fixed effects model. The *p*-value of the *LM* test rejects the null hypothesis that there is no individual random effect, so the random effects model is considered to be better than the mixed regression model. The *p*-value of the *Hausman* test also rejects the null hypothesis, so the fixed effects model is considered to be better than the random effects model. Therefore, this paper first adopts the fixed effects model to test the impact of the development of the digital economy on the health of residents. The regression results are shown in Table 3. The development of the digital economy and government fiscal revenue have a negative and significant effect on the inverse indicators of the health level of residents, and both have passed the 5% significance test. That is, the higher the level of development of the digital economy and the greater the regional fiscal revenues are, the greater the improvement of the health of residents. This is consistent with the hypothesis we proposed. The development of the digital economy provides convenience for residents to obtain more information. Some scholars believe that when people choose a certain information channel, its convenience (self-perceived channel availability) is more important than its information quality. This may be one of the reasons why the digital economy promotes the health of residents. The development of the digital economy makes it easier for people to obtain medical information and services.

**Table 3:** The result of pannel regression.

**Variables**	**Total**	**Economy_h**	**Economy_l**	**Eeconomy_h**	**Eeconomy_l**
ln_inpeo	−1.033^***^ (0.301)	−0.403^*^ (0.199)	−0.935 (0.570)	−1.240^***^ (0.255)	0.338 (0.563)
ln_govinc	−0.587^**^ (0.183)	−0.184 (0.105)	−1.769^***^ (0.449)	−0.243 (0.126)	−1.390^**^ (0.437)
ln_govoutc	−0.491 (0.602)	−0.152 (0.433)	0.675 (1.062)	0.789 (0.504)	−0.815 (1.070)
ln_yedu	1.022 (1.863)	3.604^*^ (1.766)	1.091 (2.684)	4.806^**^ (1.568)	−2.718 (3.256)
ln_labinc	1.349^*^ (0.624)	0.408 (0.460)	−0.175 (1.093)	0.181 (0.520)	0.489 (1.181)
ln_imaex	−0.334 (0.183)	−0.730^***^ (0.141)	0.314 (0.298)	−0.834^***^ (0.165)	−0.110 (0.315)
Constant	19.130^***^ (4.789)	11.176^**^ (3.545)	20.623^*^ (8.219)	3.894 (3.779)	39.967^***^ (8.760)
R-squared	0.416	0.465	0.499	0.523	0.456
*N*	510	255	255	255	255

***, **, *and* *, *respectively, indicates significance at the 1, 5, and 10% level. Standard error in parentheses*.

Analysis of heterogeneity of economic development level. There have always been significant regional development differences in the Chinese economy, and the gap between different provincial administrative regions is very large. Areas with high levels of economic development have reached the standards of developed countries, and areas with low levels of economic development are still at the level of developing countries. Different economic development levels will lead to differences in the medical services available to residents, and the health level of residents will show regional heterogeneity according to different economic development levels. This paper ranks the 30 provincial administrative regions according to their per capita GDP in 2019. The top 15 are categorised as regions with higher economic development levels (*Economy*_*H*), and the bottom 15 are categorised as regions with lower economic development levels (*Economy*_*L*). The subsample regression results are also shown in Table 3. In areas with a high level of economic development, the growth rate of Internet access has a significant inhibitory effect on the malnutrition rate of children under 5 years old, and all variables passed the significance test at the 10% level, that is, the development of the digital economy can promote the health of residents in this area. The reason may be that places with high levels of economic development have a high degree of acceptance of emerging industries such as the digital economy and big data. People who live and work in these areas have more vitality, are willing to try new things and new technologies, pay more attention to the quality of life, and pay more attention to their own physical health. In areas with a low level of economic development, people are more inclined to pursue food, clothing, and a stable life. They do not have many requirements for quality of life and lack awareness of using the Internet to make healthy investments. Therefore, the development of the digital economy has no significant impact on the health of residents in these regions. In areas with a low level of economic development, government revenues have a significant role in promoting the health of residents. The reason is that in economically underdeveloped areas, the public services and infrastructure provided by the government have a greater impact on residents in these areas, and people are more dependent on public products provided by the government.

Analysis of the heterogeneity of the development level of the digital economy. The development of China's digital economy has made the Internet and information data important factors that promote the improvement of people's lives. At this stage, China's regional digital economic development levels are different, so the impact of digital economic development on residents' health is also regionally heterogeneous. According to the “White Paper on China's Big Data Regional Development Level Evaluation (2020)” compiled by the Institute of Information Technology and Software Industry, CCID Think Tank, on the basis of the ranking of the development level of big data in 30 provincial administrative regions, this paper characterises the top 15 as regions with higher levels of digital economic development (*Eeconomy*_*H*) and the bottom 15 as regions with lower levels of digital economic development (*Eeconomy*_*L*). The subsample regression results of the digital economic development level are relatively similar to the subsample results of the economic development level; however, in areas with a high level of digital economic development, the impact of digital economic development on improving the health of residents is even greater. The possible reason for this is that residents living in areas with a high level of the digital economy are more likely to understand and use digital economy-related technologies, and their willingness to use the Internet for health investment is stronger than that in other areas.

### Threshold Effect Regression Results

The full sample and areas with low economic development levels passed the single threshold significance test and failed the double threshold significance test, that is, the number of thresholds for the best model for the country and areas with low economic development levels was 1. However, areas with a higher level of the digital economy passed the dual threshold significance test and failed the three threshold significance test, which indicates that for areas with a higher level of the digital economy, there are double thresholds. The specific results are shown in Table 4.

**Table 4:** Test for threshold.

**Sample**	**Threshold**	**F stat**	**Prob**	**BS-times**	**Crit10**	**Crit5**	**Crit1**
Total	Single	19.43^**^	0.0367	300	14.0310	18.0702	24.3904
	Double	9.69	0.2567	300	12.6030	14.4191	20.9214
Economy_H	Single	10.13	0.2200	300	14.7126	17.5901	20.4692
	Double	6.28	0.4000	300	20.0324	26.6414	43.3569
Economy_L	Single	22.09^**^	0.0433	300	17.4114	21.5934	31.3317
	Double	6.92	0.3633	300	11.3052	13.6619	18.1307
Eeconomy_H	Single	12.57^*^	0.0733	300	11.3406	14.0740	16.2549
	Double	43.32^*^	0.0800	300	36.3008	49.4610	76.0435
	Triple	3.75	0.8633	300	112.9832	140.1987	204.1399
Eeconomy_L	Single	9.07	0.3167	300	13.9155	15.7342	21.3082
	Double	10.82	0.2167	300	13.8122	19.6841	30.6827

***, **, *and* *, respectively, *indicates significance at the 1, 5, and 10% level*.

**Figure 3:**
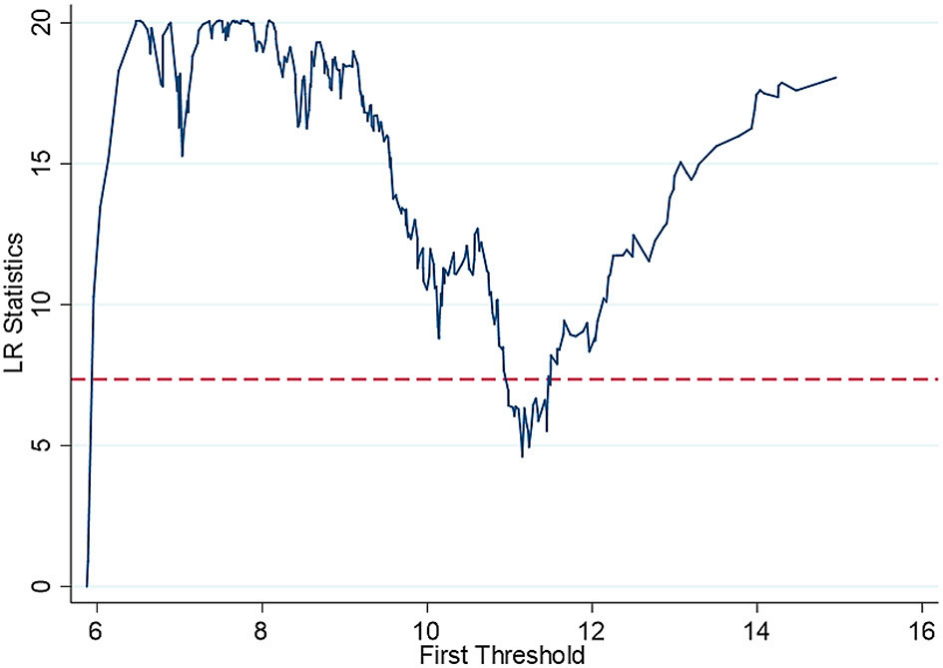
Full sample threshold regression_single threshold graph.

**Figure 4:**
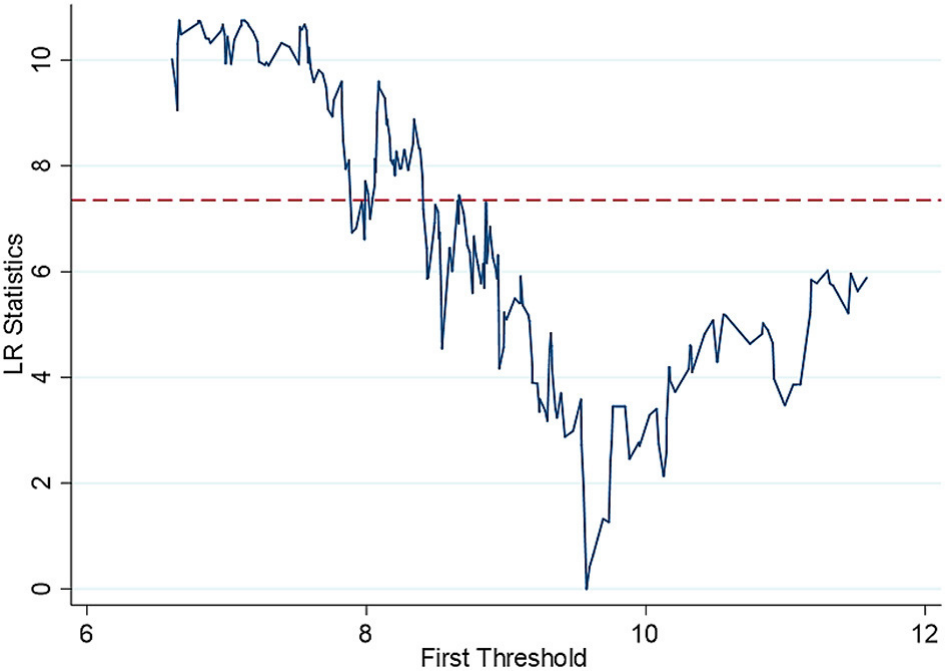
Threshold regression in economically underdeveloped areas single threshold graph.

**Figure 5:**
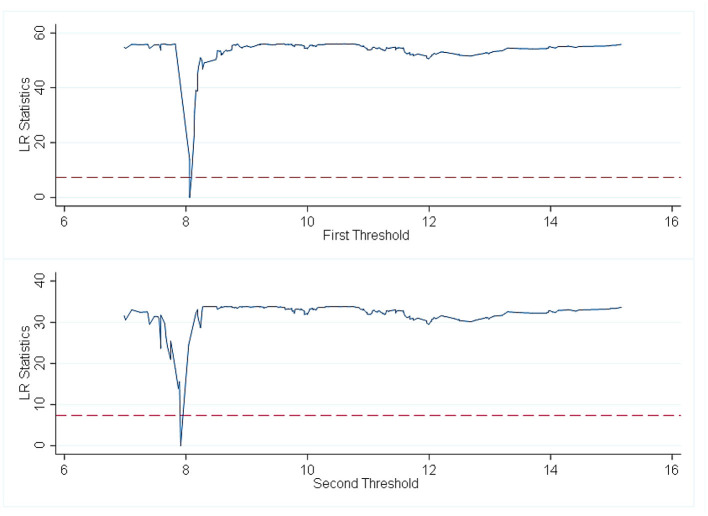
Thresholds return to high-development areas of the digital economy dual threshold diagram.

Then, the estimated value of the threshold is further obtained. The specific results are shown in Table 5 and Figures 3–5. The national threshold is 11.152, and the threshold in areas with low economic development levels is 9.5746. For areas with a high level of digital economic development, the first threshold is 7.9129, and the second threshold is 8.0887.

**Table 5:** Test for threshold value.

**Sample**	**Threshold N**	**Value**	**CI (95%)**
Total	Th-1	11.152	(10.3380, 11.1602)
Economy_L	Th-1	9.5746	(8.9689, 9.5966)
Eeconomy_H	Th-1	8.0887	(8.0521, 8.1327)
	Th-2	7.9129	(7.8923, 7.9507)

Table 6 shows that for the national sample, when the population ageing rate is lower than 11.152, there is a significant negative correlation between *ln*_*inpeo* and *denutri*, and the estimated coefficient is −0.890. When the population ageing rate is >11.152, the coefficient of the growth rate of Internet users and the rate of malnutrition of children under 5 years old remains the same; the absolute value, which is −0.816, decreases, while the impact decreases. This shows that as ageing intensifies, the development of the digital economy has reduced the effect of promoting the health of residents. This verified hypothesis two. The reason for this may be the access gap in Internet applications for the elderly in China, and the elderly have disadvantages in accessing the Internet (44).

**Table 6:** Estimated coefficients.

**Total**		**Economy_l**		**Eeconomy_H**	
ln_inpeo (oldrate < 11.152)	−0.890^**^ (0.302)	ln_inpeo (oldrate < 9.5746)	−0.714 (0.566)	ln_inpeo (oldrate < 7.9129)	−1.000^***^ (0.255)
ln_inpeo (oldrate > 11.152)	−0.816^**^ (0.306)	ln_inpeo (oldrate > 9.5746)	−0.597 (0.573)	ln_inpeo (7.9129 < ^*^ < 8.0887)	−0.792^**^ (0.276)
				ln_inpeo (oldrate > 8.0887)	−1.049^***^ (0.255)
ln_govinc	−0.562^**^ (0.181)	ln_govinc	−1.541^***^ (0.449)	ln_govinc	−0.209
					(0.123)
ln_govoutc	−0.704 (0.600)	ln_govoutc	0.343 (1.052)	ln_govoutc	0.670 (0.493)
ln_yedu	0.364 (1.856)	ln_yedu	1.207 (2.644)	ln_yedu	4.526^**^ (1.537)
ln_labinc	1.254^*^ (0.618)	ln_labinc	−0.647 (1.090)	ln_labinc	0.181 (0.504)
ln_imaex	−0.251 (0.183)	ln_imaex	0.341 (0.294)	ln_imaex	−0.851^***^ (0.160)
Constant	22.016^***^ (4.827)	Constant	23.977^**^ (8.179)	Constant	4.865 (3.684)
R-squared	0.427	R-squared	0.514	R-squared	0.552
*N*	510	*N*	255	*N*	255

***, **, *and* *, respectively, *indicates significance at the 1, 5, and 10% level. Standard error in parentheses*.

For areas with low levels of economic development, although there is a negative relationship between *ln*_*inpeo* and *denutri*, the coefficient does not pass the significance test. The existing literature has not conducted in-depth research on this issue, and this paper believes that the result may be related to historical and cultural factors in areas with low levels of economic development. Government revenues (*ln*_*govinc*) have a significant inhibitory effect on the malnutrition rate of children (*denutr*) under 5 years of age, with a coefficient of −1.541; it passes the test at the 1% significance level. This can be explained by the fact that in economically underdeveloped areas, the public services and infrastructure provided by the government have greater impacts on the residents of the area, and the public services and medical services provided by the government can improve the health of residents to a large extent.

For areas with a high level of digital economic development, when the population ageing rate is lower than 7.9129, there is a significant negative correlation between *ln*_*inpeo* and *denutri*, and the estimated coefficient is −1,000. When the population ageing rate is >7.9129 and <8.0887, the negative correlation between the growth rate of Internet users and the malnutrition rate of children under 5 years old becomes weaker and similar to the situation in the whole country; however, when the population ageing rate is >8.0887, the negative correlation between the growth rate of Internet users and the malnutrition rate of children under 5 years old increases. The reason for this is because regions with a higher level of the digital economy will take measures quicker than other regions to reduce barriers for the elderly to obtain health services through the digital economy. China vigorously promotes “Internet +” pensions and smart pensions. Areas with a higher level of the digital economy will take the lead in providing the elderly with access to and use of smartphones and provide online training for the elderly so that the elderly can learn to use the various functions of the Internet to increase the Internet usage rate of the elderly (28). The more elderly individuals use the Internet, the greater the likelihood of obtaining health investment.

### Robustness Test

In order to test the robustness of the empirical results, this paper conducts a robustness test by replacing explanatory variables and replacing the growth rate of Internet users with the Internet penetration rate. The results in Table 7 show that the development level of the digital economy has a significant impact on the health of residents. Areas with lower levels of economic development passed the single threshold significance test and failed the double threshold significance test. That is, for areas with lower levels of economic development, the best number of thresholds in the model was 1. The national sample and areas with a high level of digital economy passed the double threshold significance test but failed the three threshold significance test, which indicates that there are double thresholds for China and areas with a higher level of digital economic development. Then, the estimated value of the threshold is obtained. The specific results are shown in Table 8. The first threshold nationwide is 9.971, the second threshold is 11.152, and the threshold for areas with low economic development levels is 9.5746. For areas with a high level of digital economic development, the first threshold is 7.6932, and the second threshold is 11.9454.

**Table 7:** Robustness check_ test for threshold (change X variable).

**Sample**	**Threshold**	**Fstat**	**Prob**	**BS-times**	**Crit10**	**Crit5**	**Crit1**
Total	Single	12.85^**^	0.0333	300	9.9777	11.5380	14.5481
	Double	8.11^*^	0.0867	300	7.6325	9.2197	13.0027
	Triple	2.66	0.8133	300	7.9963	10.6030	15.6834
Economy_H	Single	5.76	0.2367	300	7.7294	9.1179	14.7549
	Double	3.14	0.4600	300	5.9933	6.6845	8.7680
Economy_L	Single	11.2^**^	0.0367	300	8.2079	9.9822	15.5595
	Double	3.93	0.4900	300	7.5140	8.6265	11.4277
Eeconomy_H	Single	4.26	0.4133	300	7.5202	9.4236	12.8711
	Double	26.09^***^	0.0067	300	7.0141	8.3461	13.2125
	Triple	3.28	0.5833	300	55.7451	89.7257	141.9737
Eeconomy_L	Single	8.13	0.2367	300	10.1657	12.3604	16.7166
	Double	9.40	0.1567	300	10.6387	11.9059	21.7479

***, **, *and* *, *respectively, indicates significance at the 1, 5, and 10% level*.

**Table 8:** Robustness check_ test for threshold value (change X variable).

**Sample**	**Threshold N**	**Value**	**CI (95%)**
Total	Th-1	9.9971	(9.5832, 10.0321)
	Th-2	11.152	(10.3327, 11.1774)
Economy_L	Th-1	9.5746	(9.2118, 9.5966)
Eeconomy_H	Th-1	11.9945	(11.9647, 12.0300)
	Th-2	7.6932	(7.6708, 8.2979)

After replacing the variables, the national sample passed the double threshold test, and the second threshold was the same as the value before the replacement variable. The single threshold passed in areas with lower levels of economic development was the same as the value before the replacement variable. The level of economic development also passed the double threshold, but it slightly increased compared to before replacing the explained variable, but it did not change significantly. The threshold regression results are shown in Table 9. For the national sample, when the population ageing rate is lower than 9.971, there is a significant negative correlation between *ln*_*inpeo* and *denutri*, and the estimated coefficient is −1.013. When the population ageing rate continues to increase, the absolute value of the estimated coefficient continues to decline. For regions with low levels of economic development, although there is a negative relationship between *ln*_*inpeo* and *denutri*, it is not significant. For areas with a high level of digital economic development, when the population ageing rate is lower than 7.6932, there is a significant negative correlation between *ln*_*inpeo* and *denutri*, with an estimated coefficient of −1.113. When the population ageing rate is >7.6932 and <11.9945, the Internet penetration rate has a reduced impact on the malnutrition rate of children under 5 years old. When the population ageing rate is >11.945, the development level of the digital economy has an increased impact on the malnutrition rate of children under 5 years old. Overall, after replacing the variables, the signs, sizes and significance levels of the core explanatory variable coefficients are very similar, and the replacement variables did not have a significant impact on the results, which indicated that the empirical results of this paper are robust.

**Table 9:** Robustness check_ estimated coefficients.

**Total**		**Economy_l**		**Eeconomy_H**	
ln_inrate (oldrate < 9.9971)	−1.013^***^ (0.255)	ln_inrate (9.5746 < oldrate)	−0.522 (0.440)	ln_inrate (oldrate < 7.6932)	−1.113^***^ (0.214)
ln_inrate (9.9971 < ^*^ < 11.152)	−0.859^***^ (0.246)	ln_inrate (oldrate < 9.5746)	−0.247 (0.451)	ln_inrate (7.6932 < ^*^ < 11.9945)	−0.924^***^ (0.233)
ln_inrate (oldrate > 11.152)	−0.713^**^ (0.255)			ln_inrate (11.9945 < oldrate)	−1.133^***^ (0.209)
ln_govinc	−0.501^**^ (0.180)	ln_govinc	−1.488^**^ (0.451)	ln_govinc	−0.170 (0.124)
ln_govoutc	−0.659 (0.574)	ln_govoutc	0.137 (0.984)	ln_govoutc	0.716 (0.488)
ln_yedu	0.099 (1.839)	ln_yedu	0.922 (2.638)	ln_yedu	4.490^**^ (1.538)
ln_labinc	0.934 (0.614)	ln_labinc	−0.829 (1.084)	ln_labinc	−0.135 (0.506)
ln_imaex	−0.167 (0.183)	ln_imaex	0.395 (0.294)	ln_imaex	−0.683^***^ (0.165)
Constant	18.952^***^ (5.052)	Constant	24.589^**^ (8.247)	Constant	−0.457 (4.035)
R-squared	0.439	R-squared	0.517	R-squared	0.550
*N*	510	*N*	255	*N*	255

***, **, *and* *, *respectively, indicates significance at the 1, 5, and 10% level. Standard error in parentheses*.

## Conclusions

This paper uses panel data to explore the threshold effect of the impact of digital economic development on the health of residents in China and various provinces under different economic development levels. In the research of this paper, the panel fixed effect regression results show that the development of the digital economy has a significant inhibitory effect on the malnutrition rate of children under 5 years old, with an impact coefficient of −1.033. After the population ageing rate is used as the threshold variable, the development of the digital economy still has a significant inhibitory effect on the malnutrition rate of children under 5 years old, but the absolute value of the coefficient significantly decreased. At the same time, in areas with a high level of digital economy development, the regression results of the double threshold value show that the absolute value of the coefficient has also declined first and then increased. This shows that the conclusions drawn without considering the issue of ageing underestimate the role of the digital economy in promoting the health of residents, and the issue of ageing is indeed an irreversible problem facing people in China and the world. These findings provide a valuable reference for policymakers on how to provide health services in a fair and efficient manner to improve the health of individuals as the digital economy continues to advance. The government can apply the digital economy to the healthcare sector. Furthermore, the advancement of the digital economy should not ignore any group, especially the elderly. The government should formulate effective Internet assistance policies for the elderly to break the barriers to the acquisition and use of the digital economy for the elderly, so that the development of the digital economy can more comprehensively promote the health of residents of all ages.

## Data Availability Statement

The original contributions presented in the study are included in the article/supplementary material, further inquiries can be directed to the corresponding author/s.

## Author Contributions

Z-TY: conceptualization and methodology. MZ: writing and editing. QG: writing—original draft. H-XW: writing and reviewing. X-HZ: software and data preparation. All authors contributed to the article and approved the submitted version.

## Conflict of Interest

The authors declare that the research was conducted in the absence of any commercial or financial relationships that could be construed as a potential conflict of interest.

## Publisher's Note

All claims expressed in this article are solely those of the authors and do not necessarily represent those of their affiliated organizations, or those of the publisher, the editors and the reviewers. Any product that may be evaluated in this article, or claim that may be made by its manufacturer, is not guaranteed or endorsed by the publisher.
